# Pilocarpine used to treat xerostomia in patients submitted to radioactive iodine therapy: a pilot study

**DOI:** 10.1590/S1808-86942010000500021

**Published:** 2015-10-22

**Authors:** Juliana Pereira Almeida, Luiz Paulo Kowalski

**Affiliations:** 1 Doctoral degree, stomatologist; 2 Livre Docente, director of the Head & Neck and Otorhinolaryngology Department, A. C. Camargo Hospital. Hospital A. C. Camargo

**Keywords:** iodine, thyroid neoplasms, xerostomia, pilocarpine.

## Abstract

**Abstract:**

Xerostomia complaint is very commonly associated to radioactive iodine therapy. Alternatives to treat this morbidity can offer better quality of life to patients with thyroid cancer submitted to adjuvant iodine therapy.

**Aim:**

to report on the experience with pilocarpine on the treatment of xerostomia in thyroid cancer patients submitted to adjuvant radioactive iodine therapy (RIT).

**Materials and methods:**

The five patients who met the inclusion criteria received 5mg of pilocarpine, 3 tid for one week. Side effects of the drug and subjective response to xerostomia complaints after treatment were evaluated.

**Design:**

it is a prospective, non-randomized study.

**Results:**

Sudoresis was the most frequent side effect of pilocarpine use, followed by fatigue and headache. Two patients reported relief of xerostomia using pilocarpine, but only one patient was able to tolerate the side effects.

**Conclusions:**

Pilocarpine seems to relieve xerostomia complaints in thyroid cancer patients because it is able to stimulate salivary flow, but the observed side effects made the patients refuse long-term therapy continuation.

## INTRODUCTION

Saliva plays an important role in the mouth. Decreased or absent flow of saliva may affect chewing, speech and swallowing, and may cause a negative impact on the patient's quality of life. The iodine-concentrating feature of salivary glands makes them potential targets during and after radioiodine therapy.^1^

Severe acute complications due to radioiodine therapy are extremely rare. Mid and long-term side effects have been well described in the literature; these effects include sialadenitis, temporary loss of taste, xerostomia and dental caries.^1^, ^2^, ^3^

Xerostomia is a dry-mouth sensation reported by patients.^4^ It may be caused by several factors, such as local or systemic diseases, drugs, radiation or chemotherapy. Alexander et al.^2^ and Caglar et al.^5^ respectively reported iodine therapy related rates of 42.9% and 54% generally one year after therapy. Our studies have shown that 11.2% of radioiodine therapy patients report xerostomia throughout the day (unpublished results).

The treatment of xerostomia is difficult; it involves relieving the symptoms by using sugar-free chewing gum, frequent hydration, saliva substitutes and sialogogues. Pilocarpine is a parasympathomimetic agent with β-adrenergic effects that stimulates cholinergic receptors on the surface of exocrine glands; it decreases the symptoms of xerostomia. Severe adverse effects are rare, but side effects such as sweating, increased urinary frequency and redness of the skin are common; these are typically moderate in intensity and last for a short period of time.^6^

The purpose of this study was to describe the efficacy and feasibility of pilocarpine for the treatment of xerostomia in patients undergoing radioiodine therapy.

## PATIENTS AND METHODS

This article is part of a large-scale retrospective cross-sectional study - with prospective data-gathering in some cases - with the following inclusion criteria: patients treated for well-differentiated thyroid carcinoma from 1997 to 2006, undergoing or not adjuvant radioiodine therapy, followed up at the Head & Neck Surgery Outpatient Unit of the A. C. Camargo Hospital, to assess possible side-effects of radioiodine therapy on salivary gland function. These criteria yielded 400 patients, of which 184 decided to participate in this study and signed a free informed consent form approved by the institutional review board of the institution (process no. 762/06). Papillary carcinoma was present in 180 patients and follicular carcinoma was present in four patients. The mean age was 49.9 years and the median age was 49 years (25-89 years). Adjuvant radioiodine therapy was done in 108 patients (58.7%). Xerostomia medication (blood pressure lowering drugs, anti-histamine medication, antidepressants) was used by 78 patients (42.4%). All patients underwent salivary gland sialometry and scintigraphy.

Sialometry was done according to the method described by Koseki et al.^7^; the total non-stimulated saliva volume was collected in a 15 ml Falcon tube for 5 minutes, and the total volume was divided by five to calculate the milliliter per minute flow. A citric acid tablet (Cewin®, Sanofi-Synthelabo, Brazil) was given to collect the total stimulated salivary flow; patients were asked to not chew the tablet, and saliva was collected as described above. The normal parameters used in this study were those published by Jensen et al. (non-stimulated flow > 0.3 ml/ min; stimulated flow > 1.5 ml/min).^4^

The following inclusion criteria were applied to verify the efficacy of pilocarpine in the treatment of xerostomia in patients submitted to radioiodine therapy: patients that underwent adjuvant radioiodine therapy simultaneously presenting 1- a complaint of xerostomia, 2- abnormal salivary flow values on sialometry, 4 and 3- salivary gland dysfunction on the visual analysis of scintigraphy. Patients with contraindications for pilocarpine, such as asthma, high blood pressure, heart diseases, and closed angle glaucoma, were excluded. Arterial blood pressure was checked before therapy. The treatment consisted of administering pilocarpine 5 mg three times a day, as recommended in the literature,^6^ mainly before meals and in the evening, during one week. Patients filled in a control questionnaire for side effects and reported their symptoms during seven days.

## RESULTS

Of 108 patients undergoing adjuvant radioiodine therapy, 65.4% (70 patients) reported xerostomia during at least part of the day, and 11.2% (12 patients) reported this symptom throughout the day. Of these 70 patients, only 9 patients met the inclusion criteria for the pilocarpine therapy protocol. The mean age of these patients was 52.1 years, and the median age was 56 years (36-65 years). The mean radioiodine irradiation dose was 150 mCi (100 - 450 mCi). Four of these nine patients were not included in the study; three patients had uncontrolled high arterial blood pressure. The data on patients included in the pilocarpine protocol are summarized in Table 1.

**Table 1:** Data on five patients that were treated with pilocarpine during a week

	Patient 1	Patient 2	Patient 3	Patient 4	Patient 5
Age	38	56	57	58	36
Dose I131 (mCi)	200	100	150	250	450
NSSF (ml/min)	0.02	0.24	0.30	0.24	0.30
SSF (ml/min)	0.42	1.38	1.10	1.16	1.50
Arterial blood pressure (mmHg)	140 × 80	120 × 80	140 × 90	120 × 80	110 × 60
Sweating	x	x	x		x
Urinary frequency	x		x		
Tearing	x		x		
Facial redness			x		
Fatigue		x	x	x	
Dizziness				x	x
Nausea				x	x
Headache		x		x	x
Increased arterial blood pressure	x				
Tachycardia	x				
Final evaluation	NT	T	NT	T	NT

NSSF - non-stimulated salivary flow; SSF - Stimulated salivary flow; NT - Not tolerated the drug because of side effects of pilocarpine; T - Tolerated the drug, the patient was able to take pilocarpine during a week and had relief of xerostomia symptoms.

The most commonly reported side effect was sweating, which was present in 4 of 5 patients. Three of 5 patients reported fatigue and headaches. Increased urinary frequency, tearing, shivering, dizziness, and nausea were present in 2 of 5 patients. One patient presented an altered arterial blood pressure and tachycardia. Two patients reported relief of xerostomia by using pilocarpine; two patients reported a significant improvement of dry mouth, but one of these patients had several side effects, and decided against long-term use of pilocarpine.

## DISCUSSION

Different from external radiotherapy, I131 radioiodine therapy does not eliminate completely salivary gland secretion; functioning parenchyma is preserved and responds to stimuli. Xerostomia is found in 11 to 54% of radioiodine therapy patients.^2^,^5^ Since well-differentiated thyroid tumors have a favorable outcome, it is essential to reduce the side effects of radioiodine therapy to preserve the quality of life of these patients.

Differently from external radiotherapy, in which several studies have demonstrated the efficacy of pilocarpine for stimulating salivary glands,^8^, ^9^, ^10^, ^11^, ^12^, ^13^, ^14^, ^15^ few studies have showed the effect of pilocarpine for relieving the symptoms of xerostomia resulting from radioiodine therapy. Silberstein^16^ published the results of a blind controlled study of 60 patients, in which pilocarpine 5 mg was used every 8 hours during a week, following radioiodine therapy, to avoid I131-associated sialadenitis. This study revealed that pilocarpine did not reduce the occurrence of radioactive iodine-associated sialadenitis. These results confirmed data published by Alexander et al.^2^ ten years ago, in which no significant difference was found between patients that were given pilocarpine during radioiodine therapy and those that were not.

To demonstrate a further use of pilocarpine, Aframian et al.^17^ published a study demonstrating the efficacy of a single 5 mg dose of pilocarpine to increase stimulated and non-stimulated salivary flow in five patients with thyroid cancer who underwent adjuvant radioiodine therapy at least three months before the study. The results showed a significantly increased stimulated and non-stimulated salivary flow in four patients; there were no changes in the systolic and diastolic blood pressure, the pulse or body temperature. Until the present, no study on the long-term use of pilocarpine for relieving radioiodine therapy-associated xerostomia has been published; the tolerability of this drug in patients has also not been evaluated.

## CONCLUSION

Our study showed that pilocarpine had no positive effects for routine use in these patients. The side effects with three daily 5 mg doses per week were poorly tolerated by patients with thyroid cancer. Thus, this medication is not recommended, as its benefits do not outweigh its side effects.

## ACKNOWLEDGEMENTS

Fundação de Amparo à Pesquisa do Estado de São Paulo - FAPESP for funding (05/60474-0 and 06/50061-2).
